# Direct air capture of CO­_2_ – topological analysis of the experimental electron density (QTAIM) of the highly insoluble carbonate salt of a 2,6-pyridine-bis(iminoguanidine), (PyBIGH_2_)(CO_3_)(H_2_O)_4_

**DOI:** 10.1107/S2052252518014616

**Published:** 2019-01-01

**Authors:** Christopher G. Gianopoulos, Zhijie Chua, Vladimir V. Zhurov, Charles A. Seipp, Xiaoping Wang, Radu Custelcean, A. Alan Pinkerton

**Affiliations:** a Department of Chemistry, University of Toledo, Toledo, OH 43606, USA; b Chemical Sciences Division, Oak Ridge National Laboratory, Oak Ridge, TN 37831, USA; c Neutron Scattering Division, Oak Ridge National Laboratory, Oak Ridge, TN 37831, USA

**Keywords:** carbon capture, guanidine, X-ray diffraction, neutron diffraction, charge density, topological analysis, crystal engineering, intermolecular interactions, hydrogen bonding, environmental chemistry

## Abstract

Chemical bonding and all intermolecular interactions in the highly insoluble carbonate salt of a 2,6-pyridine-bis(iminoguanidine), (PyBIGH_2_)(CO_3_)(H_2_O)_4_, recently employed in the direct air capture of CO_2_ via crystallization, have been analyzed within the framework of the quantum theory of atoms in molecules (QTAIM) based on the experimental electron density derived from X-ray diffraction data obtained at 20 K. Accurate hydrogen positions were included based on an analogous neutron diffraction study at 100 K. Topological features of the covalent bonds demonstrate the presence of multiple bonds of various orders within the PyBIGH_2_^2+^ cation. Strong hydrogen bonds define ribbons comprising carbonate anions and water molecules. These ribbons are linked to stacks of essentially planar dications via hydrogen bonds from the guanidinium moieties and an additional one to the pyridine nitro­gen. The linking hydrogen bonds are approximately perpendicular to the anion–water ribbons. The observation of these putative interactions provided motivation to characterize them by topological analysis of the total electron density. Thus, all hydrogen bonds have been characterized by the properties of their (3,−1) bond critical points. Weaker interactions between the PyBIGH_2_^2+^ cations have similarly been characterized. Integrated atomic charges are also reported. A small amount of cocrystallized hydroxide ion (∼2%) was also detected in both the X-ray and neutron data, and included in the multipole model for the electron-density refinement. The small amount of additional H^+^ required for charge balance was not detected in either the X-ray or the neutron data. The results are discussed in the context of the unusually low aqueous solubility of (PyBIGH_2_)(CO_3_)(H_2_O)_4_ and its ability to sequester atmospheric CO_2_.

## Abstract 2

Low-temperature X-ray and neutron diffraction experiments are used to characterize the electron-density distribution and intermolecular interactions in the insoluble carbonate salt of a 2,6-pyridine-bis(iminoguanidine), a potential CO_2_ sequestering agent. The importance of the hydrogen-bonding ability of the cation to the anion–water structure is discussed.

## 1. Introduction   

Given the strong connection between climate change and the greenhouse gases in the atmosphere, developing new methods to reduce their concentration in the air and alleviate global warming is of major importance. Besides curbing the use of fossil fuels, one strategy for limiting the increase in the atmospheric CO­_2_ concentration is based on carbon capture and storage (CCS) (Lackner, 2003 ▸; Reiner, 2016 ▸). Significant progress has been made in employing CCS at point sources of CO_2_ emission, such as coal- or gas-fired power plants. However, this approach does not address the problem of diffuse sources of CO­_2_, such as households and transportation, which are responsible for approximately 50% of total greenhouse gas emissions. Most climate change mitigation scenarios aiming to limit global warming to 2°C or less now include implementation of negative emissions technologies (NETs) that target net reductions of the atmospheric CO_2_ concentration, currently ∼408 p.p.m. One promising approach among various NETs under consideration is direct air capture (DAC), a process that removes CO_2_ from the air by engineered chemical reactions (Keith, 2009 ▸; Lackner *et al.*, 2012 ▸; Sanz-Pérez *et al.*, 2016 ▸; Keith *et al.*, 2018 ▸).

In a recent study, an aqueous solution of a 2,6-pyridine-bis(iminoguanidine), PyBIG [the displayed resonance form is based on the reported crystal structure (Seipp *et al.*, 2017 ▸; Brethomé *et al.*, 2018 ▸)], was found to efficiently absorb CO_2_ from the atmosphere and convert it into the crystalline carbonate salt (PyBIGH_2_)(CO_3_)(H_2_O)_4_ (Seipp *et al.*, 2017 ▸; Brethomé *et al.*, 2018 ▸). The main driver for this reaction is the extremely low aqueous solubility of the carbonate salt (*K*_sp_ = 1.0 × 10^−9^), comparable to CaCO_3_ (3.7–8.7 × 10^−9^), which pushes the overall equilibrium towards the carbonate formation despite the very low concentration of CO_2_ in the air. After filtration, crystalline (PyBIGH_2_)(CO­_3_)(H_2_O)_4_ is mildly heated at 120°C to release the CO_2_ (which can be sent to storage) and regenerate the PyBIG sorbent for reuse in another DAC cycle. Thus, this crystallization-based approach offers the prospect for energy-efficient DAC technology, provided the synthesis of PyBIG and the overall CO_2_ capture process can be optimized and scaled up cost effectively.[Chem scheme1] 
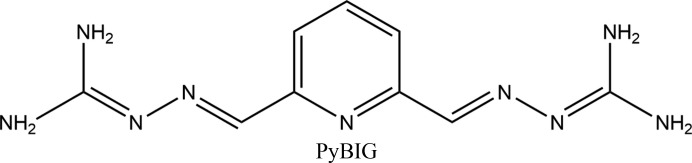


The previously reported X-ray crystal structure of (PyBIGH_2_)(CO­_3_)(H_2_O)_4_ showed that the hydrated carbonate salt comprises an elaborate hydrogen-bonded network involving the carbonate anion, guanidinium cations, water molecules and the pyridine N atom (Seipp *et al.*, 2017 ▸; Brethomé *et al.*, 2018 ▸). However, the precise geometrical parameters and energetics of the hydrogen bonds and other intermolecular interactions present in this structure, which presumably play important roles in the unusually low aqueous solubility of (PyBIGH_2_)(CO­_3_)(H_2_O)_4_, have yet to be determined. To this end, as reported in this article, we have determined precisely all hydrogen positions from neutron diffraction data, which provides an accurate geometrical description of all the hydrogen bonds present. The interaction energies of these hydrogen bonds have been estimated from a topological analysis of the electron density, as determined from extremely accurate high-resolution X-ray diffraction data. At the same time, we have characterized all of the covalent bonds and the integrated atomic charges within the framework of the quantum theory of atoms in molecules (QTAIM) (Bader, 1994 ▸).

## 2. Experimental   

### 2.1. Data collection and reduction   

#### 2.1.1. X-ray experiment   

Colorless crystals of (PyBIGH_2_)(CO_3_)(H_2_O)_4_ were obtained by slow reaction of an aqueous solution of PyBIG with atmospheric CO_2_. A single crystal (0.31 × 0.20 × 0.15 mm) was subsequently mounted with oil on top of a thin-walled glass capillary, and cooled to 20 K with an open-flow helium cryostat (Hardie *et al.*, 1998 ▸; Kirschbaum *et al.*, 1999 ▸). X-ray diffraction measurements were performed with a Rigaku diffractometer equipped with a Mo rotating anode generator operating at 50 kV and 300 mA (ULTRAX-18 Mo *K*α, curved graphite monochromator) and using a RAPID-II cylindrical image-plate detector. To obtain highly redundant data, runs collecting 30 × 6° ω scans were performed at χ = 0°, ϕ = 0 and 180°, and at χ = 40°, ϕ = 0, 90, 180 and 270°. These runs were augmented by collecting an analogous set with ω offset by 3°. Thus, frames were overlapped by a half-frame width to improve scaling and allow for the omission of partial and overlapping reflections. An exposure time of 180 s per image was chosen to maximize he scattering power and avoid saturation of the strongest reflections.

The collected data were indexed and reflection positions predicted using the program *HKL2000* (Otwinowski & Minor, 1997 ▸). Data were integrated with the program *VIIPP*, applying an image-plate flood-field correction, and with background and reflection profiles averaged over the whole data set, as described previously (Zhurova *et al.*, 1999 ▸, 2008 ▸; Zhurov & Pinkerton, 2013 ▸). Partial and overlapped reflections were rejected during the integration. The effects of absorption (μ = 0.122 mm^−1^) and thermal diffuse scattering at 20 K were considered to be negligible. Additional outliers were identified and removed manually through equivalence comparison to minimize outlier contamination. This is particularly important for removing errors from multiple scattering, and for identifying previously unidentified partial and overlapping reflections. This resulted in 1.41% of measured data (2730 out of 193 867 reflections) being additionally rejected prior to merging and scaling of the data in the space group 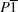 with the program *SORTAV* (Blessing, 1995 ▸, 1987 ▸, 1997 ▸). Corrections of reflection intensities for λ/2 contamination were also made (Kirschbaum *et al.*, 1997 ▸; Gianopoulos *et al.*, 2017 ▸). Other experimental details are listed in Table 1 ▸.

#### 2.1.2. Neutron experiment   

Data from a single-crystal plate (2.01 × 1.50 × 0.37 mm) prepared as above were obtained using the *TOPAZ* single-crystal neutron time-of-flight (TOF) Laue diffractometer (Jogl *et al.*, 2011 ▸; Schultz *et al.*, 2014 ▸) at the Spallation Neutron Source (SNS) at Oak Ridge National Laboratory. The diffractometer is equipped with 24 detectors, each with an active area of 15 × 15 cm, arranged on a near-spherical detector array tank. The initial moderator-to-sample flight path is 18 m and the sample-to-detector distances vary in the range 39–46 cm. The total path length of 18.4 m and the SNS pulse rate of 60 Hz provides a wavelength bandwidth of 3.6 Å. The crystal was mounted on a MiTeGen loop using cyano­acrylate glue and cooled to 100 K for data collection. A total of 47 crystal orientations optimized with *CrystalPlan* software (Zikovsky *et al.*, 2011 ▸) were used to ensure better than 95% coverage of a hemisphere of reciprocal space. Data were displayed, auto-indexed and integrated using the suite of algorithms in *Mantid* (Arnold *et al.*, 2014 ▸). The raw Bragg intensities were obtained using the three-dimensional ellipsoidal *Q*-space integration method (Schultz *et al.*, 1984 ▸). Data reduction including the neutron TOF spectrum, Lorentz, and detector efficiency corrections were carried out with the *ANVRED3* program (Schultz *et al.*, 1984 ▸). A Gaussian numerical absorption correction was applied with μ = 0.1506 + 0.1027λ mm^−1^. The reduced data were saved in *SHELX* HKLF2 format, in which the neutron wavelength for each reflection was recorded separately.

### 2.2. Refinements   

The crystal structure of PyBIG carbonate was reported previously (Seipp *et al.*, 2017 ▸; Brethomé *et al.*, 2018 ▸) and we have preserved the setting of the unit cell and the atom numbering used in that work. Based on our experimental neutron data, the crystal structure was re-refined within the *SHELXTL* program suite (Sheldrick, 2015 ▸) using the previously reported structure as the starting model. All atoms were refined using anisotropic thermal motion. An initial least-squares refinement based on the X-ray data was also carried out (*SHELXTL*). Anisotropic thermal motion was considered for all non-hydrogen atoms, and the hydrogen atoms were refined isotropically. From this starting point, a multipole refinement based on the Hansen–Coppens pseudo-atom formalism (Hansen & Coppens, 1978 ▸) [equation (1)], as implemented in the *MoPro* program package (Jelsch *et al.*, 2005 ▸), using the Volkov and co-workers relativistic data bank (Volkov *et al.*, 2006 ▸), was performed,
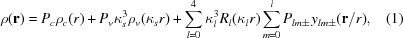
where ρ_*c*_ and ρ_*v*_ are spherical core and valence densities normalized to one electron, *P*_*c*_ and *P*_*v*_ are the core and spherical valence populations, respectively, *R_l_* represents normalized Slater-type radial functions, *y_lm_* are real angular spherical harmonics, and *P_lm_* refers to the multipole population of the *m*th term of the *l*th order. The κ_*s*_ and κ_*l*_ terms are expansion–contraction coefficients for the spherical and multipolar valence densities, respectively.

All ‘heavy’ atoms were refined to the hexadecapole level, while the hydrogen atoms were refined up to dipoles plus the bond-directed quadrupole, with C—H, N—H and O—H distances constrained to the values obtained from the neutron study. In the initial stages of refinement, chemical constraints for similar atoms were applied; however, these constraints were gradually released, and the final model was refined unconstrained (24 refined multipole populations for each ‘heavy’ atom and 4 refined multipole populations for hydrogen atoms), with the exception of κ parameters (see below). The molecular electroneutrality requirement was applied throughout for the total structure. This allowed for charge transfer among the charged species rather than constraining their formal charge. The expansion–contraction parameters κ_*s*_ and κ_*l*_ for the non-hydrogen atoms were refined in ten groups according to their chemical equivalence, while κ_*s*_ and κ_*l*_ for hydrogen atoms were set to 1.2. The final description of the anisotropic thermal motion for the hydrogen atoms was obtained from *SHADE-3.1* (Madsen, 2006 ▸).

The residual map calculated after the multipole refinement still had one unidentified peak significantly above background. Examination of the neutron residual showed the same small feature along with a negative neighbor (Figs. S8 and S9). These features were identified as a small number of cocrystallized hydroxide ions. The refined occupancies were 0.017 (2) from the X-ray data (IAM model) and 0.022 (2) from neutrons. No evidence was found in the neutron data for the H^+^ required for charge balance, hence we assume that it is disordered over the available oxygen and nitro­gen sites. The final multipole refinement then included a variable occupancy for the contribution from a spherically modeled oxygen atom.

Topological analysis of the total electron density was carried out with the program packages *MoPro* (Jelsch *et al.*, 2005 ▸), *XDPROP* (Volkov *et al.*, 2006 ▸) and *WinXPRO* (Stash & Tsirelson, 2002 ▸, 2005 ▸).

### 2.3. Evaluation of X-ray data quality   

Analysis of statistical measures of data and multipole model quality have been deposited; all suggest excellent data and an excellent model. Averaged ratios (in 0.05 Å^−1^ bins) of observed and calculated structure factors (Fig. S1) as well as the normal probability plot (Fig. S2) indicate good model fitting for the whole sinθ/λ range. The residual electron density maps (Fig. S3) are low (ρ_min/max_ = −0.186/0.272 e Å^−3^, calculated for the complete data set) and featureless as confirmed by a fractal dimension plot (Fig. S4). The total electron density was non-negative everywhere.

## 3. Results and discussion   

### 3.1. Structure   

**Figure 1:**
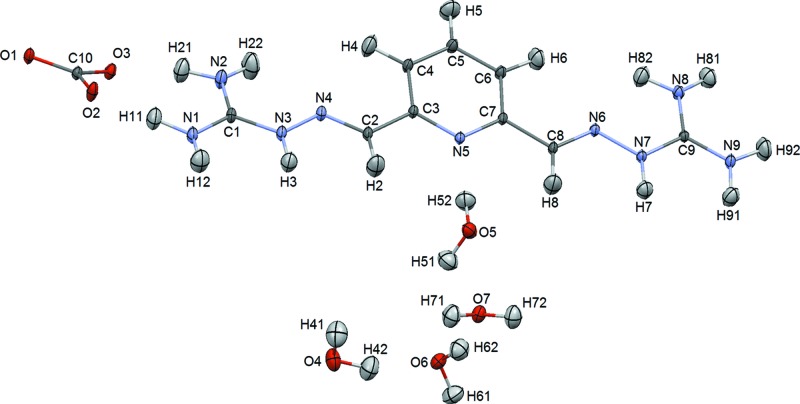
The asymmetric unit of PyBIG carbonate tetrahydrate, as determined from 100 K neutron diffraction data, showing the atom numbering. Displacement ellipsoids are at the 50% probability level (Macrae *et al.*, 2008 ▸).

**Figure 2:**
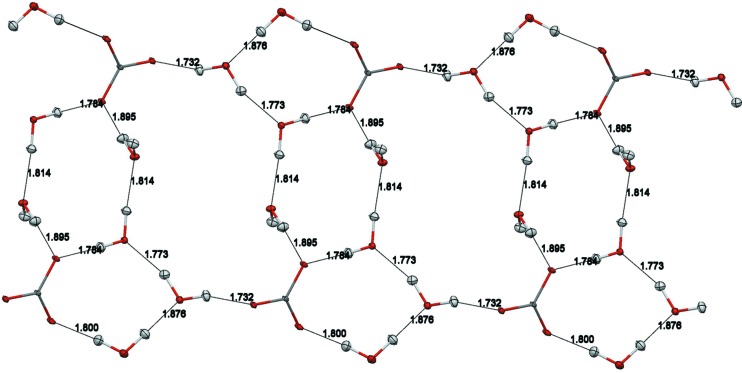
Potential hydrogen bonds in the anion–water ribbons. The figure is based on the neutron structure, with displacement ellipsoids at the 20% probability level and distances in Å (Macrae *et al.*, 2008 ▸). Color scheme – oxygen, red; carbon, dark gray; hydrogen, light gray.

**Figure 3:**
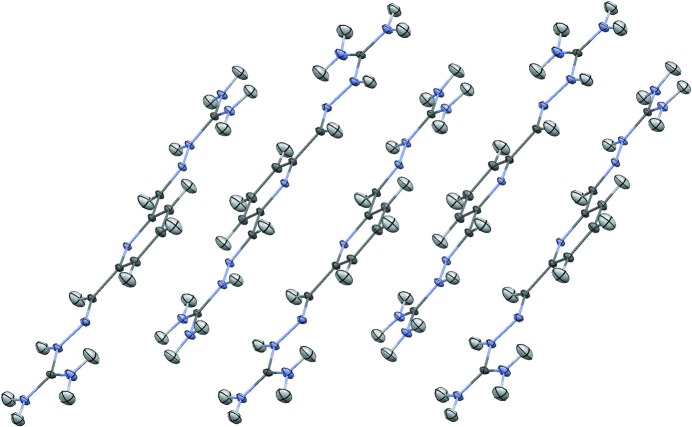
The stacking of PyBIGH_2_^2+^ cations. The figure is based on the neutron structure, with displacement ellipsoids drawn at the 20% probability level (Macrae *et al.*, 2008 ▸). Color scheme: nitrogen, blue; carbon, dark gray; hydrogen, light gray.

As reported previously (Seipp *et al.*, 2017 ▸; Brethomé *et al.*, 2018 ▸), the structure is made up of essentially planar PyBIGH_2_^2+^ dications, carbonate anions and four water molecules. The asymmetric unit and the atom labeling are shown in Fig. 1 ▸. The anions and water molecules form a ribbon (Fig. 2 ▸), and based on distance criteria, we may already propose that this structure is strongly hydrogen bonded, as shown in the figure. The cations form stacks (Fig. 3 ▸) that are approximately perpendicular to the plane of the anion–water ribbons.

The anion–water ribbon is canted at an angle of ∼23.6° above and below the *ac* plane, and extends about 1.91 Å above and below the plane (Fig. S5). The ribbon has a maximal thickness of ∼1.49 Å (on the basis of heavy atoms), while the H52 atom is oriented nearly perpendicular (81.1°) to the mean plane of the ribbon. While each ribbon extends infinitely along the *a* axis, the width of each ribbon is ∼11.82 Å, about 2 Å shorter than the length of the *c* axis. The distance between nearest neighbors on different adjacent ribbons is approximately 4.2 Å and gives rise to a channel between neighboring ribbons. In this context, it is unsurprising that the sites of the partially occupied hydroxide ions fall in this cavity and are suggestive of a stabilizing interaction between neighboring ribbons (Fig. S5). On the basis of the neutron diffraction results, the nearest hydroxide HO⋯H distance is ∼1.90 Å, while the nearest HO—H⋯OH distance is ∼1.73 Å. The cations lie roughly above and below the *ac* plane containing the extended network of ribbons. When viewed along the [101] direction it becomes clear that the nearest cations are all hydrogen-bond donors to the water–anion ribbons and form linear arrays along the [101] vector, nearly in the (¼ 0 ¼) plane. Slightly further from the ribbons are cation arrays (along [101]) wherein the hydrogen-bond-accepting pyridine N5 atom is oriented towards the ribbon (Figs. S6 and S7).

**Figure 4:**
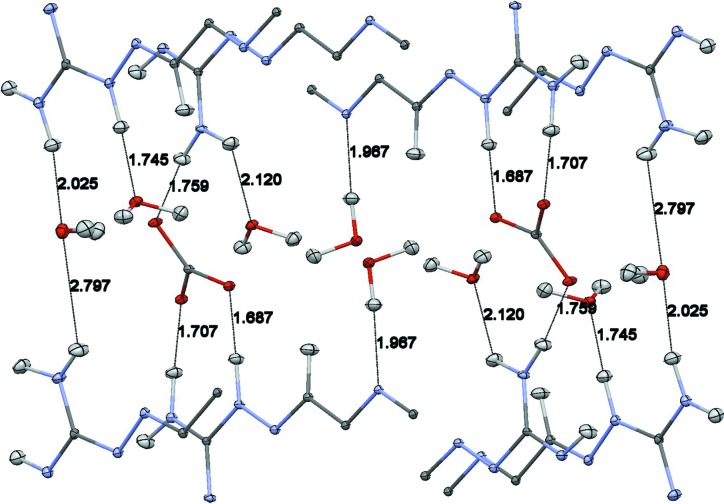
Potential hydrogen bonds between the anion–water ribbon and neighboring cations. All cations have been truncated to enhance the visibility of the hydrogen bonds. The figure is based on the neutron structure, with displacement ellipsoids drawn at the 20% proabability level and distances in Å (Macrae *et al.*, 2008 ▸). Color scheme: oxygen, red; nitrogen, blue; carbon, dark gray; hydrogen, light gray.

The distances shown in Fig. 4 ▸ suggest strong hydrogen bonds between the guanidinium hydrogen atoms and a variety of oxygen atoms, as well as a water hydrogen bonded to the pyridine nitro­gen. Note that the cations in Fig. 4 ▸ have been truncated to emphasize the possible hydrogen-bond interactions. It is also clear from Fig. 3 ▸ that there is potential for additional interactions between the π-systems of neighboring cation sheets as they are only separated by ∼3.2 Å. The observation of these putative noncovalent interactions provided much of the motivation to characterize them by topological analysis of the total electron density.

## 4. Electron density   

### 4.1. Atomic charges   

**Figure 5:**
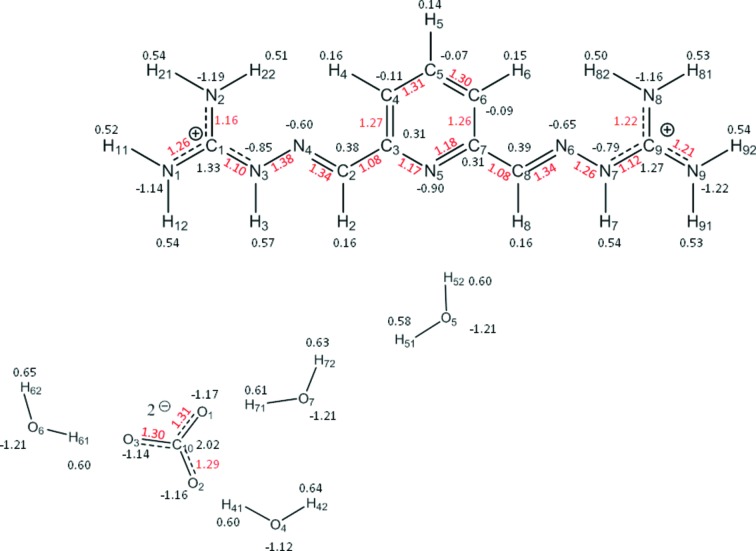
Integrated atomic charges (black) and topological bond orders (red).

The integrated charges of the atomic basins delimited by the zero-flux surfaces and their volumes are listed in Table 2 ▸. For convenience, the charges are also reported in Fig. 5 ▸. The accuracy of the integration for each atom was confirmed by a small value of the integrated Laplacian (Lagrangian). The atomic charges sum to zero as required; however, the total charge of the cation and anion differ from the formal value of 2.0, indicating significant charge transfer. Concomitantly, all water molecules are close to neutral, and the disordered OH group contributes a small amount of negative charge. The sum of the atomic volumes is close to the unit-cell volume per asymmetric unit with an error of ∼0.2%. All oxygen atoms have significant negative charges of similar magnitudes, whether in the anion or in the water molecules. The nitro­gens are all strongly negative, and may be differentiated according to their type (NH_2_ < N_pyridine_ < NH < N_imino_). The carbon atoms have a wide range of mainly positive charges that strongly correlate with their environment (C_carbonate_ > C_guanidine_ > C_imino_ > C_pyridine_ – the latter being slightly negative). As expected, the hydrogen atoms are all strongly positive and again may be grouped by type (H_2_O > NH > CH).

**Figure 6:**
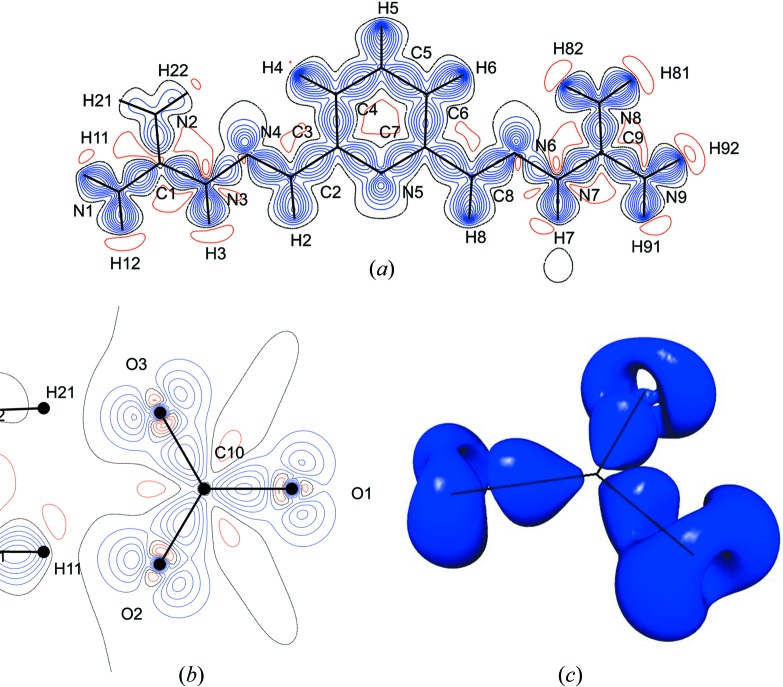
Deformation density (*a*) in the plane of the dication and (*b*) in the plane of the anion. Blue contours are positive density and red ones are negative. The contour level is 0.10 e Å^−3^. (*c*) The anion deformation density iso-surface at 0.15 e Å^−3^.

The deformation density in the plane of the dication is mapped in Fig. 6 ▸(*a*) and clearly shows a significant concentration of electron density in all of the covalent bonds, as well as the presence of lone pairs on the imino and pyridine nitro­gen atoms. The covalent bonding density is also well represented for the anion and for the water molecules. Again, the expected lone-pair regions on the oxygen atoms are also well defined.

**Figure 7:**
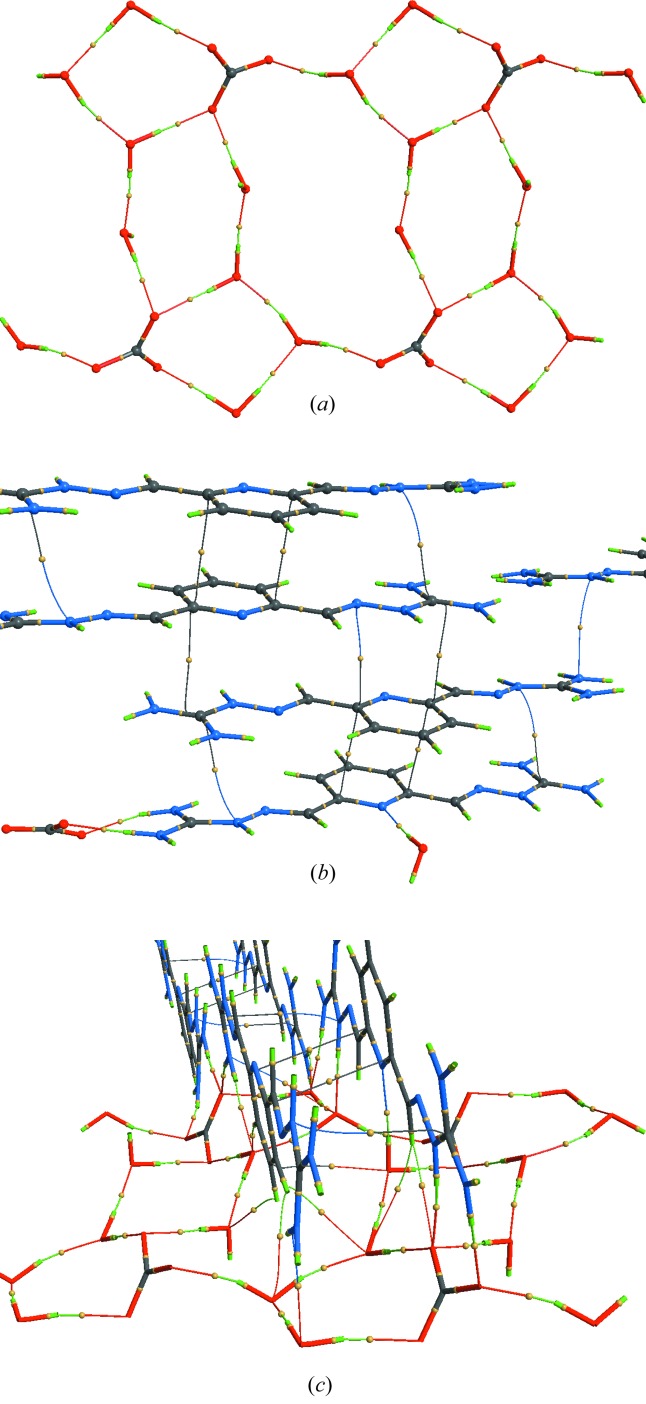
Bond paths and critical points (yellow spheres) for (*a*) all covalent bonds and intermolecular hydrogen bonds in the carbonate–water ribbons, for (*b*) all covalent bonds in the dication and cation–cation stacking interactions, and (*c*) selected bond paths and critical points linking the cationic stacks and anionic ribbons. Color scheme: C, black; N, blue; O, red; H, green.

More complete information on the nature of the bonding may be obtained from a topological analysis of the total electron density (Fig. 7 ▸ and Table 3 ▸). All (3,−1) critical points for the covalent bonds in the dication, the carbonate anion, and selected water molecules are indicated by yellow spheres in Figs. 7 ▸(*a*) and 7(*b*). Their characteristics are tabulated in Table 3 ▸. All covalent bonds have significant electron density at the critical point, with negative values of the Laplacian as required. Of particular interest is the extent of electron delocalization (π bonding) in the essentially planar dication. In general, all bonds in the molecular skeleton are short, with significant ellipticities at the critical points indicating important π-character. Complementary information on the nature of these bonds may be obtained from the topological bond orders as defined by *n*_topo_ = *a* + *b*λ_3_ + *c*(λ_1_ + λ_2_) + *d*ρ_CP_, where ρ is the electron density at the critical point, λ_1,2,3_ are obtained from the Hessian matrix, and the coefficients (*a*, *b*, *c*, *d*) were taken from the literature (Howard & Lamarche, 2003 ▸; Tsirelson *et al.*, 2006 ▸, 2007 ▸; Bartashevich *et al.*, 2011 ▸). The bond orders for the skeleton of the cation, which range from 1.078 to 1.382 and show close to twofold molecular symmetry in their value, further indicate the delocalized π-character of the C—C and C—N bonds (Table 3 ▸ and Fig. 5 ▸). The strongest bonds are those involving the imino atoms N4 and N6, whereas the weakest are the substituted guanidinium C—N bonds (C1—N3 and C9—N7) and the substituents of the pyridine ring (C2—C3 and C7—C8). The bond orders of the N—H bonds are significantly lower than those of C—H, corresponding to the higher positive charges on the H(N) atoms compared with H(C).

Although Fig. 6 ▸(*b*) implies well resolved lone pairs on all the oxygen atoms of the carbonate anion, these are actually cuts through a doughnut-like charge distribution as shown by the iso-surface plot in Fig. 6 ▸(*c*). As expected, the carbonate anion is strongly covalently bonded, and the charge distribution is highly polarized, the oxygen atoms carrying high negative charges and the carbon atom being strongly positive.

### 4.2. Closed-shell interactions   

The most important noncovalent interactions in this structure are the hydrogen bonds, which range in strength from modest to strong. All bond paths have been identified from the topology of the electron density, and their (3,−1) bond critical points characterized (Table 4 ▸ and Fig. 7 ▸). Every hydrogen atom in the structure bonds to a neighboring oxygen or to the pyridine nitrogen (N5) except H22, which only bonds to the partially occupied OH group. All carbonate oxygen atoms accept three hydrogen bonds and all water oxygen atoms accept two. The only nitro­gen atom that accepts a hydrogen bond is the pyridine N5 atom.

We have estimated the dissociation energy of each hydrogen bond based on the topological analysis and assuming the validity of the relationship by Espinosa *et al.* (1998 ▸, 1999 ▸). The dissociation energies of the most important hydrogen bonds range from modest (∼14 kJ mol^−1^) to strong (∼66 kJ mol^−1^). From the 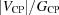 criterion (Espinosa *et al.*, 2002 ▸), although these are still closed-shell interactions, the stronger ones are well within the so-called ‘transit’ region, 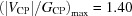.

As shown in the figures, we may divide the hydrogen bonds into two sets, one which defines the carbonate–water ribbons and another roughly perpendicular to the first linking the anionic ribbons and cationic stacks. We recognize that this is an artificial classification as the two types have similar energies; however, we believe that it provides some insight into the design criteria for new cations to provide such insoluble materials.

**Figure 8:**
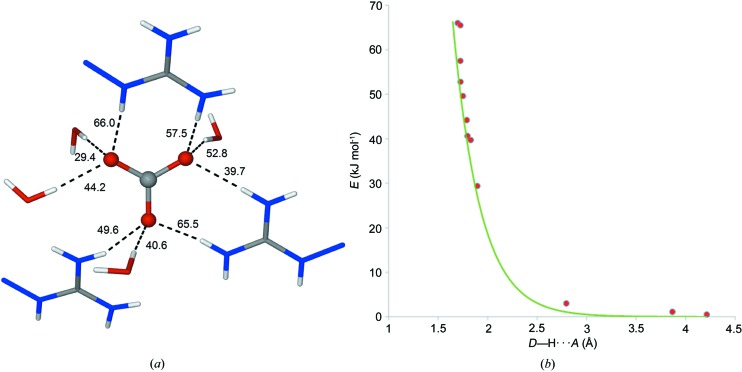
Hydrogen bonding involving the carbonate anion. (*a*) Carbonate ‘binding’ by five guanidinium and four water hydrogen bonds, with estimated dissociation energies in kJ mol^−1^. (*b*) Observed correlation between hydrogen-bond energies and H⋯O contact distances; red points are from our experiment and the green line is from the work by Espinosa *et al.* (1998 ▸).

As expected, the strongest hydrogen bonds involve the carbonate anion, which accepts a total of nine hydrogen bonds, five from the guanidinium groups and four from the water molecules (Fig. 8 ▸ *a*), as well as three much weaker interactions. The correlation between the hydrogen-bond energies and the observed H⋯O contact distances (Fig. 8 ▸ *b*) may be fitted to an exponential curve as anticipated from the derivation of the Espinosa relationship. The estimated carbonate ‘binding’ energy from all hydrogen bonds and the three weaker interactions amount to −449.9 kJ mol^−1^. Notably, a large fraction of the carbonate ‘binding’ energy (−167 kJ mol^−1^, 37.1%) comes from hydrogen bonding to water. Clearly, the water molecules of hydration play an important role in the stability, and thereby the low aqueous solubility of (PyBIGH_2_)(CO­_3_)(H_2_O)_4_ crystals, by providing a total of −441.9 kJ mol^−1^ in hydrogen-bonding energy. The strong hydrogen bonding of carbonate and water in these crystals is needed to partially compensate for the large free energy of dehydration of the anion (1315 kJ mol^−1^) (Marcus, 1991 ▸) involved in the crystallization of PyBIGH_2_(CO­_3_)(H_2_O)_4_. Additionally, the lattice energy, consisting of electrostatic as well as other interactions (vide infra), must also contribute to the low aqueous solubility of these crystals.

A complete topological analysis of the electron density reveals a number of additional bond paths, suggesting much weaker interactions. Although not rigorously justified, extrapolating the Espinosa *et al.* (1998 ▸, 1999 ▸) relationship suggests that dissociation energies for these additional interactions are all <5 kJ mol^−1^ (Table 4 ▸). By definition, a bond path must begin and end at a nucleus; however, due to the close face-to-face proximity of the planar cations, many of these interactions may be better described as π–π interactions.

### 4.3. Lattice energy and electrostatic interactions   

It is well known that high lattice energies tend to lower the solubility of crystalline compounds: 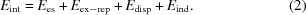
Although not the only contributor to the lattice energy [*E*_int_, equation (2)], electrostatic interactions (*E*_es_) tend to dominate this quantity in ionic crystals (Coppens, 1997 ▸; Volkov *et al.*, 2006 ▸). Determination of the exchange–repulsion and dispersion to the total interaction energy are method dependent and, hence, unreliable. However, the electrostatic term may be obtained from the multipole expansion of the electron density using the methodology proposed by Volkov *et al.* (2004 ▸). Thus, we have determined the electrostatic crystal binding energy for (PyBIGH_2_)(CO_3_)(H_2_O)_4_ to be −583 kJ mol^−1^. Although this may seem modest for an ionic compound, we have noted significant charge transfer between the cation and the anion, and the cationic charge is highly delocalized.

## 5. Conclusions   

This article reports both a neutron diffraction study and a high-resolution X-ray diffraction study of a highly insoluble carbonate salt formed by crystallization of a guanidine compound with atmospheric CO_2_. Both diffraction methods confirm the presence of a small amount of cocrystallized hydroxide ion. The accurately determined topological properties of the electron density characterize the delocalized nature of the bonding in the planar cation, as well as two well developed strong hydrogen-bonding schemes, one defining an anion–water ribbon, and the other essentially orthogonal to the anion–water ‘plane’, linking the anionic ribbons to the cationic stacks. The carbonate anions are strongly hydrogen bonded in these crystals, which likely contributes to the extremely low aqueous solubility of this salt. The water molecules of hydration, hydrogen bonded to the carbonate anions and the guanidinium cations, also play an important role in the stability of these crystals. While the intermolecular interactions are dominated by strong hydrogen bonds, a number of supplementary weaker interactions have been characterized. Although their bond paths have been identified by their nuclear attractors, many may better be characterized as π–π interactions. The electrostatic contribution to the lattice energy is relatively modest due to both charge transfer and charge delocalization.

## Supplementary Material

Crystal structure: contains datablock(s) global, xray, neutron. DOI: 10.1107/S2052252518014616/lt5014sup1.cif

supplementary figures and tables. DOI: 10.1107/S2052252518014616/lt5014sup2.pdf

CCDC references: 1873555, 1880876

## Figures and Tables

**Table 1:** Experimental details CIFs for both experiments are provided in the supporting information.

	X-ray	Neutron
Empirical formula	C_9_H_15_N_9_^2+^·CO_3_^2−^·4H_2_O	C_9_H_15_N_9_^2+^·CO_3_^2−^·4H_2_O
Crystal size (mm)	0.31 × 0.20 × 0.15	2.01 × 1.50 × 0.37
Crystal shape	Plate	Plate
Wavelength (Å)	0.71073	0.40–3.39 (TOF)
Crystal system	Triclinic	Triclinic
Temperature (K)	20	100
Space group	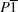	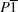
*a* (Å)	8.2090(2)	8.2420 (2)
*b* (Å)	8.5762 (2)	8.6011 (3)
*c* (Å)	13.8676 (4)	13.8821 (4)
α (°)	72.591 (2)	72.792 (3)
β (°)	78.815 (2)	78.998 (3)
γ (°)	71.0422 (17)	70.789 (2)
*V* (Å^3^), *Z*	875.97 (4), 2	882.92 (5), 2
Density (g cm^−3^)	1.447	1.436
μ (mm^−1^)	0.122	0.1506 + 0.1027λ
(sinθ/λ)_max_ (Å^−1^)	1.30	2.45
Reflections integrated	189348	44971
*R*_int_, average data multiplicity	0.027, 9.1	0.0963, 5.6
Completeness: sinθ/λ < 0.76 Å^−1^, all data (%)	99.0/80.0	91.4
Independent reflections	25805	7955
Used reflections	18335 [*I* > 3σ(*I*)]	7955
		
Spherical refinement		
*R*_1_[*F*, *I* > 2σ(*I*)], *wR*_2_(*F* ^2^), GOF	0.028, 0.082, 1.055	0.034,0.065, 1.128
Δρ_min/max_ for X-rays (e Å^−3^), for neutrons (fm Å^−3^)	−0.33/0.79	−1.185/1.104
		
Multipole refinement		
No. of parameters	1232	
*R*_1_[*F*, *I* > 3σ(*I*)], *wR*_2_(*F* ^2^), GOF	0.018, 0.020, 1.115	
Δρ_min_/_max_ (e Å^−3^), sinθ/λ < 1.3 Å^−1^	−0.186, 0.272	
Weighting scheme: *a*, *b* †	0.0038, 0.0038	

†
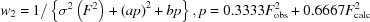

**Table 2:** Integrated atomic charges (*q*) and volumes (Ω)

Atom	*q* (e)	Ω (Å^3^)	Atom	*q* (e)	Ω (Å^3^)
PyBIG cation			Carbonate		
N1	−1.14	17.06	C10	2.02	4.43
H12	0.54	2.33	O1	−1.17	17.90
H11	0.52	2.08	O2	−1.16	17.97
C1	1.33	5.32	O3	−1.14	17.08
N2	−1.19	19.93	Total	−1.45	57.38
H22	0.51	2.25			
H21	0.54	2.15	Water		
C2	0.38	10.28	O4	−1.12	18.46
H2	0.16	7.04	H42	0.64	1.33
N3	−0.85	12.51	H41	0.60	1.70
H3	0.57	1.83	Total	0.12	21.49
C3	0.31	7.98			
N4	−0.60	14.72	Water		
C4	−0.11	11.65	O5	−1.21	20.95
H4	0.16	6.12	H52	0.60	1.94
C5	−0.07	11.61	H51	0.58	2.16
H5	0.14	6.89	Total	−0.04	25.05
N5	−0.90	13.88			
C6	−0.09	11.98	Water		
H6	0.15	6.16	O6	−1.21	18.00
N6	−0.65	13.02	H62	0.65	1.57
C7	0.31	8.03	H61	0.60	1.75
N7	−0.79	12.29	Total	0.04	21.32
H7	0.54	1.87			
C8	0.39	10.32	Water		
H8	0.16	7.01	O7	−1.21	19.83
N8	−1.16	16.23	H72	0.63	1.76
H82	0.50	2.94	H71	0.61	1.74
H81	0.53	2.23	Total	0.03	23.33
C9	1.27	5.09			
N9	−1.22	17.51	Hydroxyl		
H92	0.54	3.26	O8	−0.01	14.82
H91	0.53	1.98			
Total	1.32	275.55	Total/total	0.01	438.94
					
			Cell volume/2		437.99

**Table 3:** Characteristics of covalent bond critical points in PyBIG carbonate tetrahydrate ε = λ_1_/λ_2_ – 1; *n*_topo_ = *a* + *b*λ_3_ + *c*(λ_1_ + λ_2_) + *d*ρ_CP_ (Howard & Lamarche, 2003 ▸; Tsirelson *et al.*, 2006 ▸, 2007 ▸; Bartashevich *et al.*, 2011 ▸); complete tables of bond critical-point properties have been included in the supporting information.

Atom 1	Atom 2	ρ(r) (e Å^−3^)	∇^2^ρ(r) (e Å^−5^)	*R*_ij_ (Å)	ε	*n*_topo_
PyBIG						
C1	N1	2.492	−26.400	1.325	0.167	1.260
C1	N2	2.455	−30.350	1.327	0.219	1.157
C1	N3	2.372	−28.050	1.352	0.173	1.104
C2	C3	1.909	−16.050	1.468	0.102	1.084
C2	H2	1.832	−19.780	1.095	0.046	0.894
C2	N4	2.637	−31.840	1.286	0.219	1.342
C3	C4	2.139	−20.070	1.398	0.192	1.265
C3	N5	2.349	−22.860	1.347	0.113	1.169
C4	C5	2.162	−20.020	1.391	0.138	1.314
C4	H4	1.878	−20.420	1.088	0.035	0.928
C5	C6	2.168	−20.320	1.388	0.163	1.304
C5	H5	1.870	−20.250	1.086	0.022	0.927
C6	C7	2.111	−19.480	1.402	0.184	1.258
C6	H6	1.892	−20.850	1.085	0.028	0.921
C7	C8	1.899	−15.870	1.470	0.117	1.078
C7	N5	2.374	−23.750	1.345	0.112	1.183
C8	H8	1.836	−19.560	1.096	0.055	0.912
C8	N6	2.637	−31.870	1.284	0.192	1.342
C9	N7	2.352	−25.500	1.358	0.186	1.121
C9	N8	2.486	−28.670	1.322	0.209	1.215
C9	N9	2.453	−27.400	1.326	0.188	1.212
N1	H11	2.134	−31.950	1.005	0.036	0.635
N1	H12	2.129	−32.370	1.037	0.042	0.689
N2	H21	2.091	−31.280	1.029	0.037	0.659
N2	H22	2.155	−30.160	1.013	0.045	0.730
N3	H3	2.031	−31.380	1.057	0.043	0.626
N3	N4	2.375	−7.047	1.362	0.084	1.382
N6	N7	2.406	−7.880	1.357	0.074	1.367
N7	H7	2.039	−30.490	1.041	0.047	0.622
N8	H81	2.092	−30.960	1.030	0.025	0.657
N8	H82	2.203	−32.860	0.998	0.037	0.687
N9	H91	2.124	−31.530	1.015	0.043	0.663
N9	H92	2.079	−30.100	1.045	0.041	0.695
Carbonate						
C10	O1	2.506	−30.380	1.289	0.111	1.309
C10	O2	2.476	−30.030	1.289	0.113	1.289
C10	O3	2.467	−29.830	1.292	0.128	1.302
Waters						
O4	H41	2.274	−36.720	0.980	0.046	0.595
O4	H42	2.254	−41.790	0.971	0.024	0.550
O5	H51	2.223	−33.490	0.967	0.016	0.449
O5	H52	2.248	−37.030	0.973	0.009	0.528
O6	H61	2.205	−36.630	0.981	0.022	0.505
O6	H62	2.210	−41.440	0.980	0.005	0.526
O7	H71	2.243	−38.560	0.973	0.005	0.516
O7	H72	2.159	−37.750	0.984	0.020	0.472

**Table 4:** Characteristics of bond critical points for closed shell intermolecular interactions Complete tables of bond critical point properties are provided in the supporting information.

Atom 1	Atom 2	Symmetry†	ρ(r) (e Å^−3^)	∇^2^ρ(r) (e Å^−5^)	*R*_ij_ (Å)‡	*D*_e_ (kJ mol^−1^)	Acceptor group/donor group
O1	H7	46401	0.358	2.093	1.698	66.0	Carbonate/imino
O2	H11	55501	0.354	2.208	1.725	65.5	Carbonate/guan
O3	H91	46401	0.317	2.527	1.725	57.5	Carbonate/guan
O6	H3	65501	0.299	2.466	1.724	53.1	Water/imino
O3	H61	66502	0.296	2.590	1.727	52.8	Carbonate/water
O2	H81	45401	0.279	2.700	1.751	49.6	Carbonate/guan
O1	H71	56502	0.266	2.170	1.790	44.2	Carbonate/water
O7	H62	55501	0.258	2.544	1.766	44.2	Water/water
O2	H41	56502	0.251	2.074	1.798	40.6	Carbonate/water
O5	H72	65602	0.237	2.561	1.797	40.0	Water/water
O3	H21	55501	0.244	2.180	1.830	39.7	Carbonate/guan
O6	H42	55501	0.200	2.171	1.868	31.3	Water/water
O1	H51	56502	0.187	2.292	1.897	29.4	Carbonate/water
N5	H52	55501	0.201	1.608	1.963	28.8	Pyridine/water
O4	H12	65501	0.154	1.475	2.003	20.5	Water/guan
O7	H82	66602	0.108	1.718	2.115	15.5	Water/guan
O4	H92	55401	0.092	1.708	2.129	13.6	Water/guan
O7	H6	66602	0.055	0.527	2.561	4.9	Water/pyridine
O5	H5	54501	0.047	0.647	2.647	4.9	Water/pyridine
N1	N3	56502	0.051	0.562	3.256	4.8	Guan/imino
C3	C9	66602	0.057	0.463	3.280	4.7	Pyridine/guan
C7	N6	66602	0.054	0.498	3.267	4.7	Pyridine/imino
N3	C9	56602	0.049	0.458	3.398	4.1	Imino/guan
O5	C5	56602	0.042	0.454	3.216	3.6	Water/pyridine
O7	H4	64501	0.034	0.511	2.807	3.4	Water/pyridine
O6	H4	64501	0.028	0.513	2.807	3.2	Water/pyridine
O1	H8	46401	0.030	0.453	2.796	3.0	Carbonate/imino
O4	N2	64501	0.026	0.477	3.366	2.9	Water/guan
O4	N2	66502	0.021	0.277	3.550	1.8	Water/guan
O6	N8	66602	0.019	0.282	3.535	1.7	Wate/guan
O7	H8	65602	0.020	0.235	3.138	1.5	Water/imino
O1	C5	57502	0.015	0.166	3.867	1.1	Carbonate/pyridine
O1	N2	47502	0.008	0.087	4.214	0.5	Carbonate/guan

† *ORTEP* symmetry codes for Atom 2.

‡ The shorter values for the intermolecular distances compared with those reported for the neutron study are caused by lattice contraction at lower temperature.
