# Supplementary material for: Direct air capture of CO­2 – topological analysis of the experimental electron density (QTAIM) of the highly insoluble carbonate salt of a 2,6-pyridine-bis(iminoguanidine), (PyBIGH2)(CO3)(H2O)4
Source: IUCrJ. 2019 Jan 1;6(Pt 1):56–65. doi: 10.1107/S2052252518014616 (PMC6327188; doi:10.1107/S2052252518014616)
Supplement: Supplementary file 2 [file m-06-00056-sup2.pdf]

IUCrJ

Volume 6 (2019)

Supporting information for article:

Direct air capture of CO2 – topological analysis of the experimental electron density (QTAIM) of the highly insoluble carbonate salt of a 2,6-pyridine-bis(iminoguanidine), (PyBIGH2)(CO3)(H2O)4

Christopher G. Gianopoulos, Zhijie Chua, Vladimir V. Zhurov, Charles A. Seipp, Xiaoping Wang, Radu Custelcean and A. Alan Pinkerton

SUPPLEMENTARY MATERIAL

CONTENTS

Fig. S1 Averaged ratios (in 0.05 Å^{-1} bins) of observed and calculated structure factors	S3
Fig. S2 Normal probability plot	S3
Fig. S3 Residual electron density maps	S4
Fig. S4 Fractal dimension plot and probability distribution histogram	S5
Fig. S5 Packing of anion-water ribbons.	S6
Fig. S6, S7 Packing of cations about the anion-water ribbons.	S7
Fig. S8 Residual density map from preliminary neutron refinement.	S8
Fig. S9 Residual density map from preliminary multipole refinement.	S9
Table S1 Properties of bond critical points for covalent bonds	S10
Table S2 Properties of bond critical points for intermolecular interactions	S10
Table S3-S4 Reflection statistics	S14

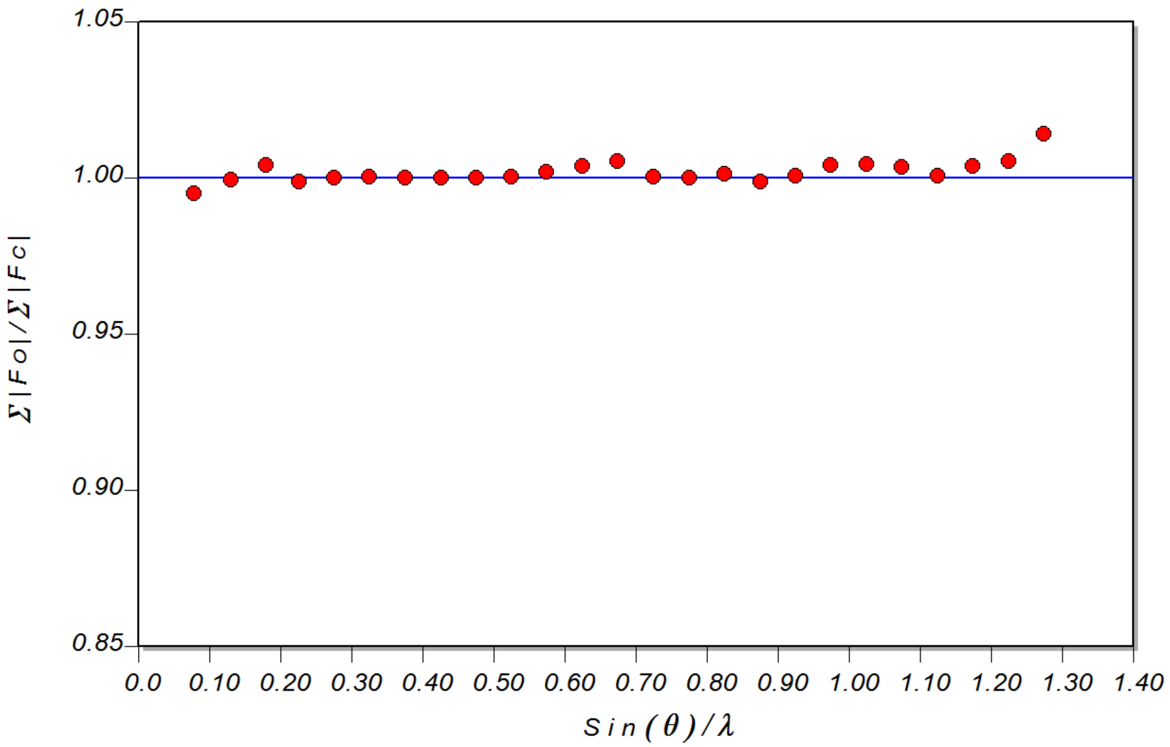

Fig. S1 Averaged ratios (in 0.05 $Å^{-1}$ bins) of observed and calculated structure factors

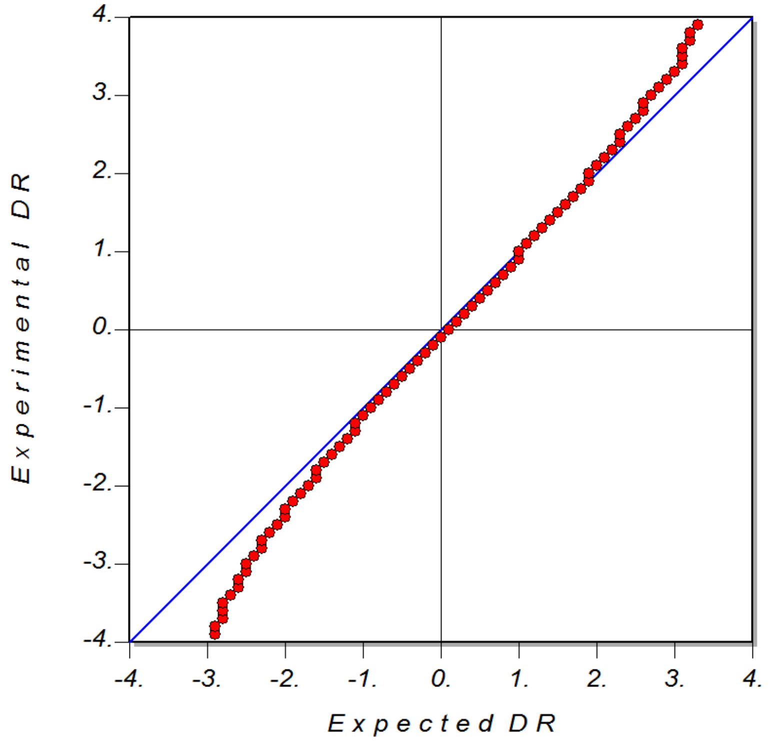

Fig. S2 Normal probability plot

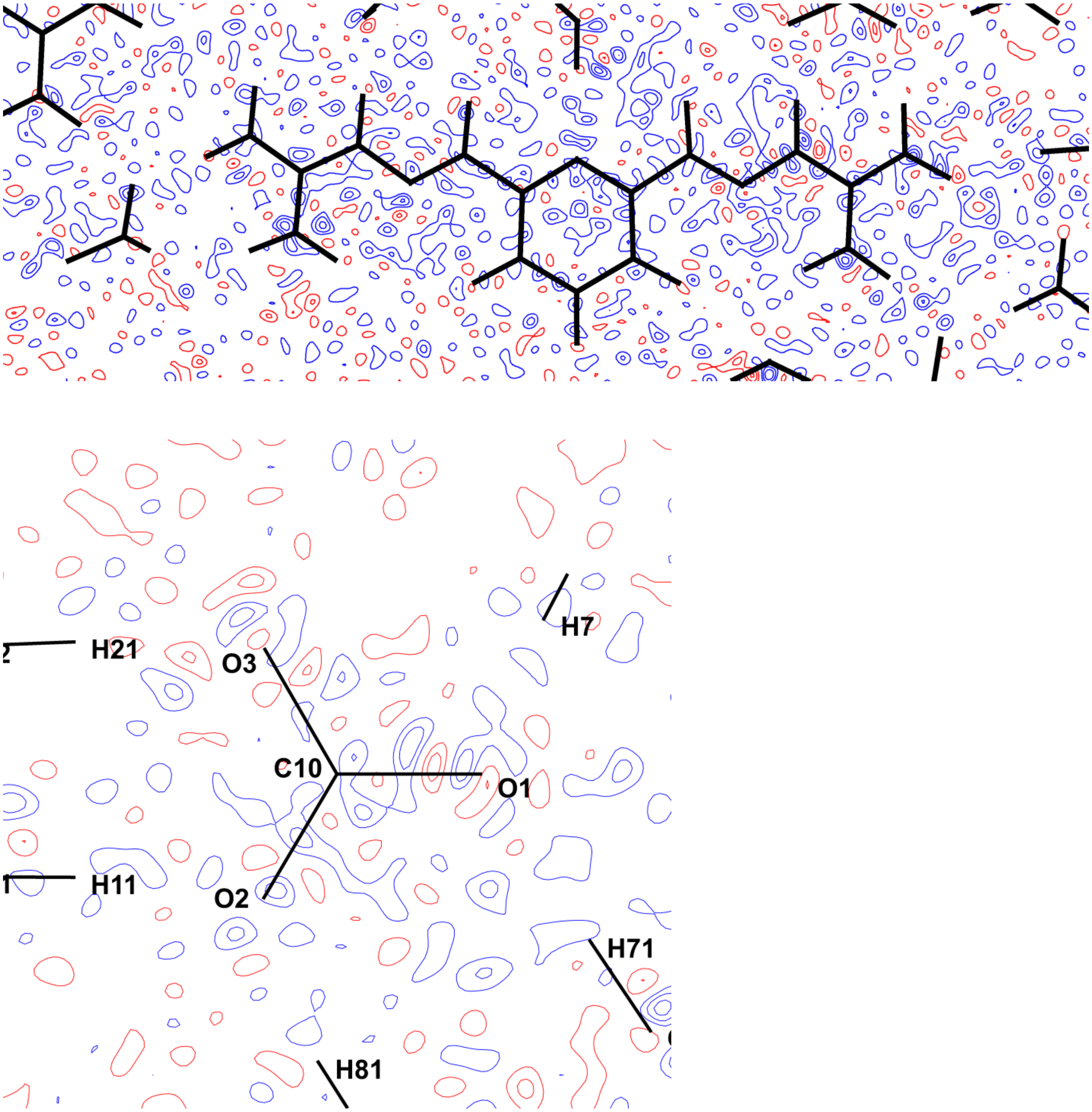

Fig. S3 Residual electron density maps in the plane of the cation (top) and anion (bottom). Contours at the 0.05 e/ang3 level; blue, positive – red, negative.

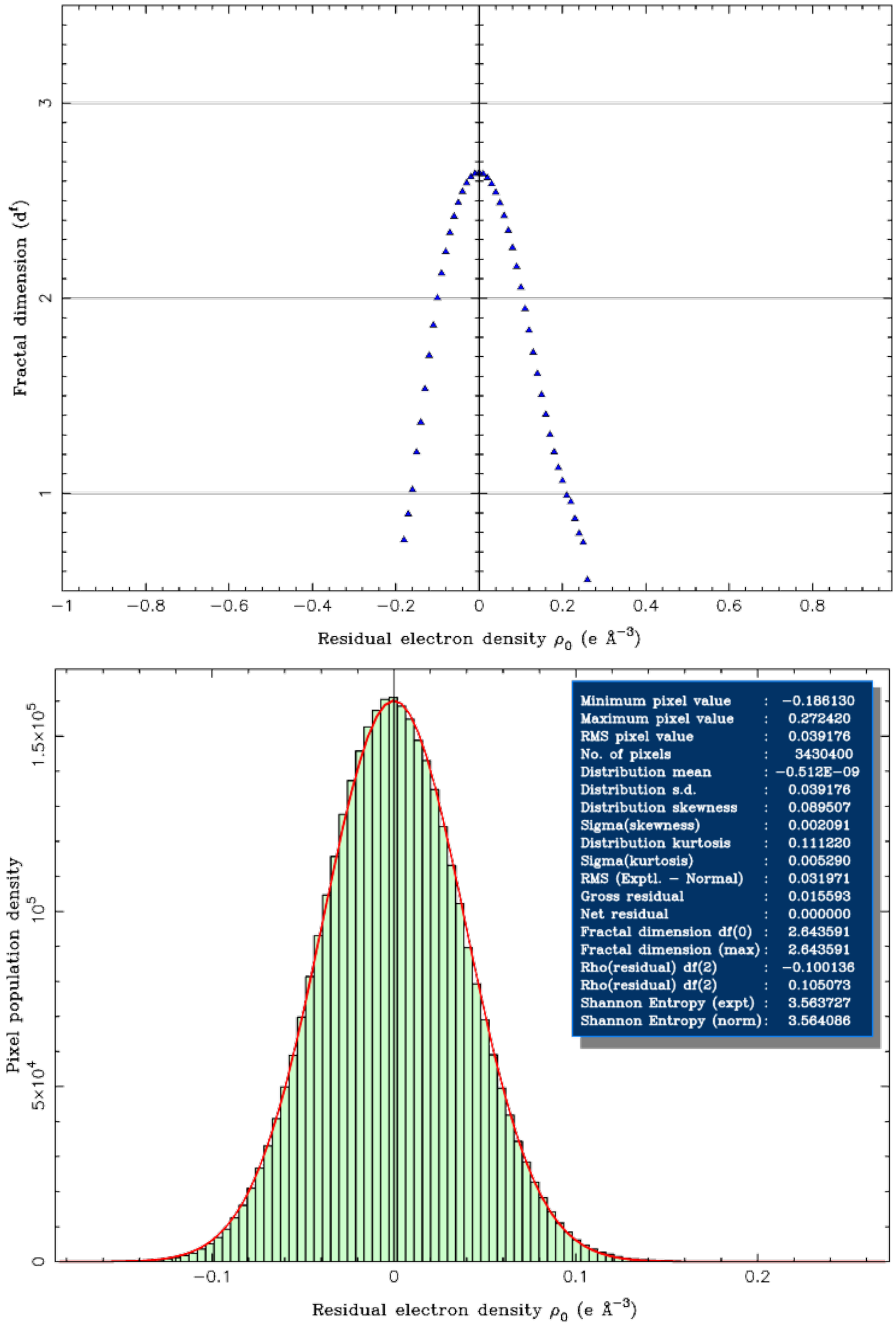

Fig. S4 Fractal dimension plot and probability distribution histogram

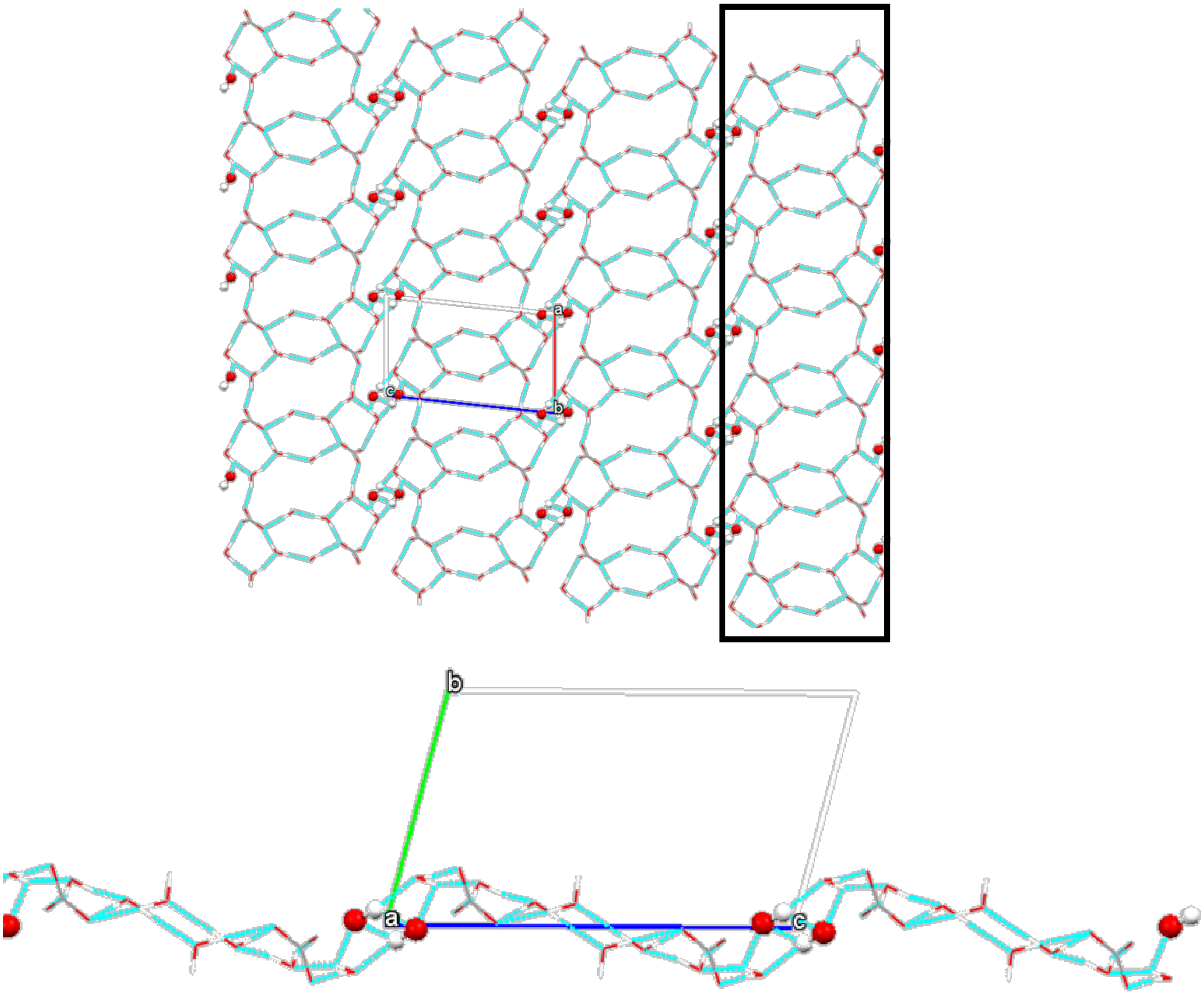

Fig. S5. Packing of anion-water ribbons. In the above figure, four ribbons are depicted from the perspective of looking down the *b*-axis. The ribbons extend infinitely along the *a*-axis, a portion of one these ribbons is highlighted by the black box. The disordered OH^- ions are depicted as balls, while the ordered anion water-ribbons are depicted in wireframe, blue lines denote short contacts (H-bonds). The bottom figure illustrates the canting of the ribbons above and below the *ac*-plane as viewed from the perspective of looking down the *a*-axis.

The anion-water ribbon is canted at an angle of ~23.6° above and below the *ac*-plane, and extends about 1.91 Å above and below the plane. In this context it is unsurprising that the sites of the partially occupied hydroxide ions fall in this cavity and are suggestive of a stabilizing interaction between neighboring ribbons

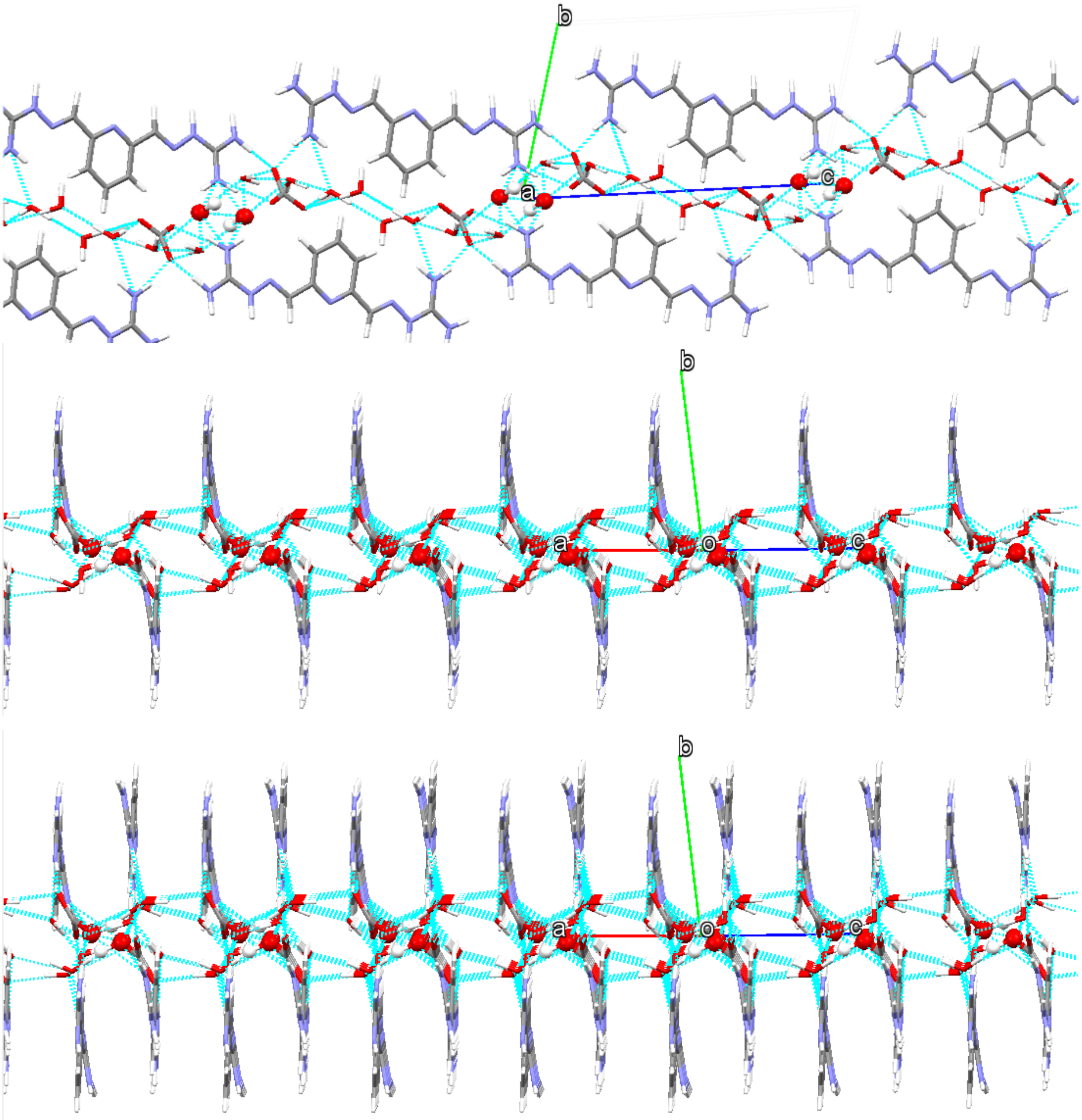

Fig. S6a, S6b, S7 Packing of cations about the anion-water ribbons. There are two differing types of interactions between the cation arrays and the anion-water ribbons. The nearest cation arrays (which pack infinitely along the [101] direction) to the ribbons participate as H-bond donors (see above and middle figures which provide two perspectives of the same diagram, and wherein all cation Npy point away from the anion-water ribbon). Slightly further away from the ribbons are cation arrays wherein the H-bond accepting pyridine N5 atom is oriented towards the ribbon. Both figures are from the perspective of looking down the [101] direction. Cations are depicted as sticks, the anion-water ribbons are depicted in wireframe and the disordered OH^- ions are shown as balls.

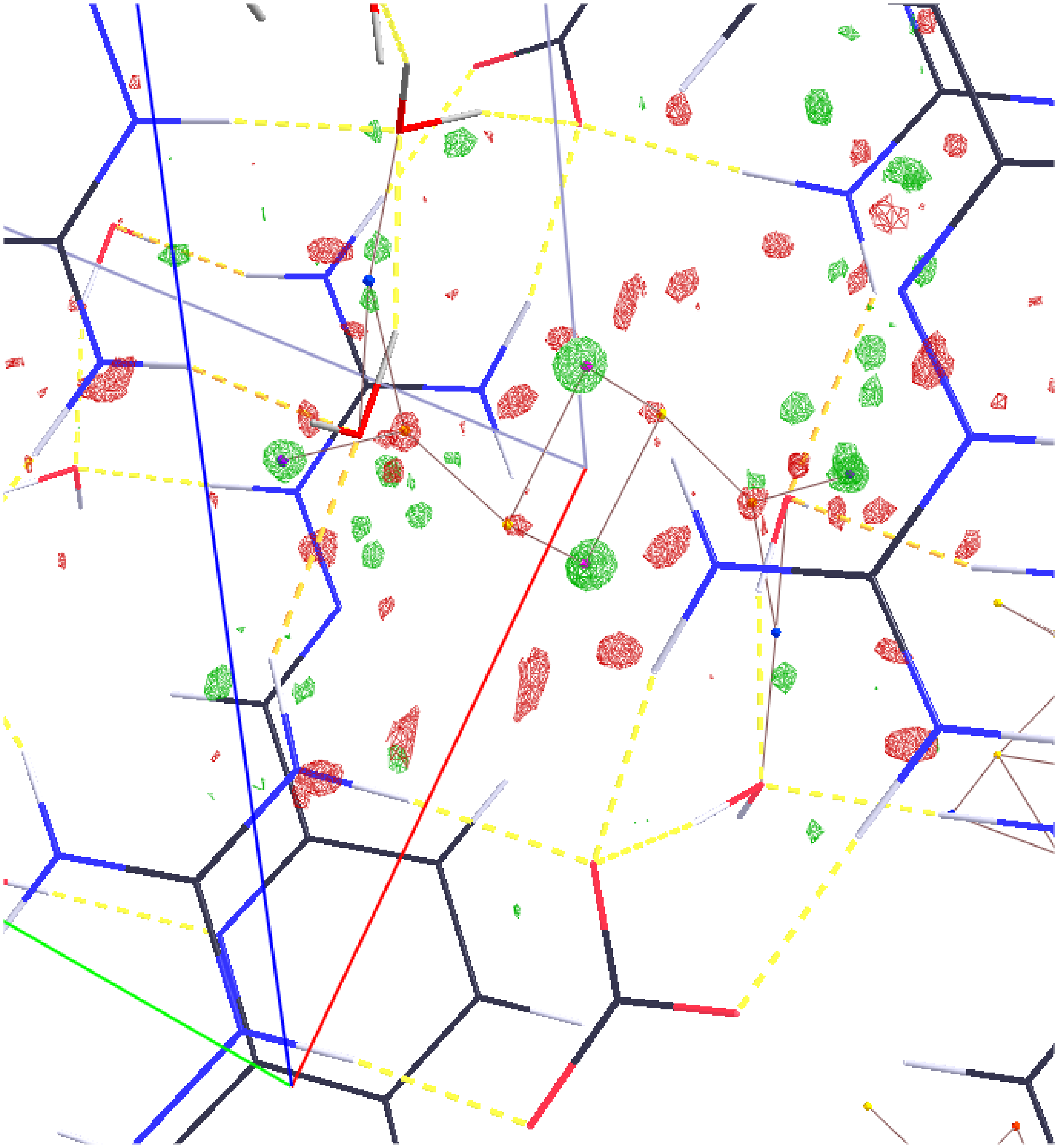

Figure S8. Residual density map from preliminary neutron refinement excluding the partially occupied OH group. The maximum is 1.40 fm Å^{-3}, nearby minimum -0.78 fm Å^{-3} and 1-sigma level 0.205 fm Å^{-3}.

Maximum coordinates; peak height: 0.9747 -0.0200 0.0794; 1.40 fm Å^{-3}

Minimum coordinates; peak height: 1.0980 -0.0779 0.0280; -0.78 fm Å^{-3}

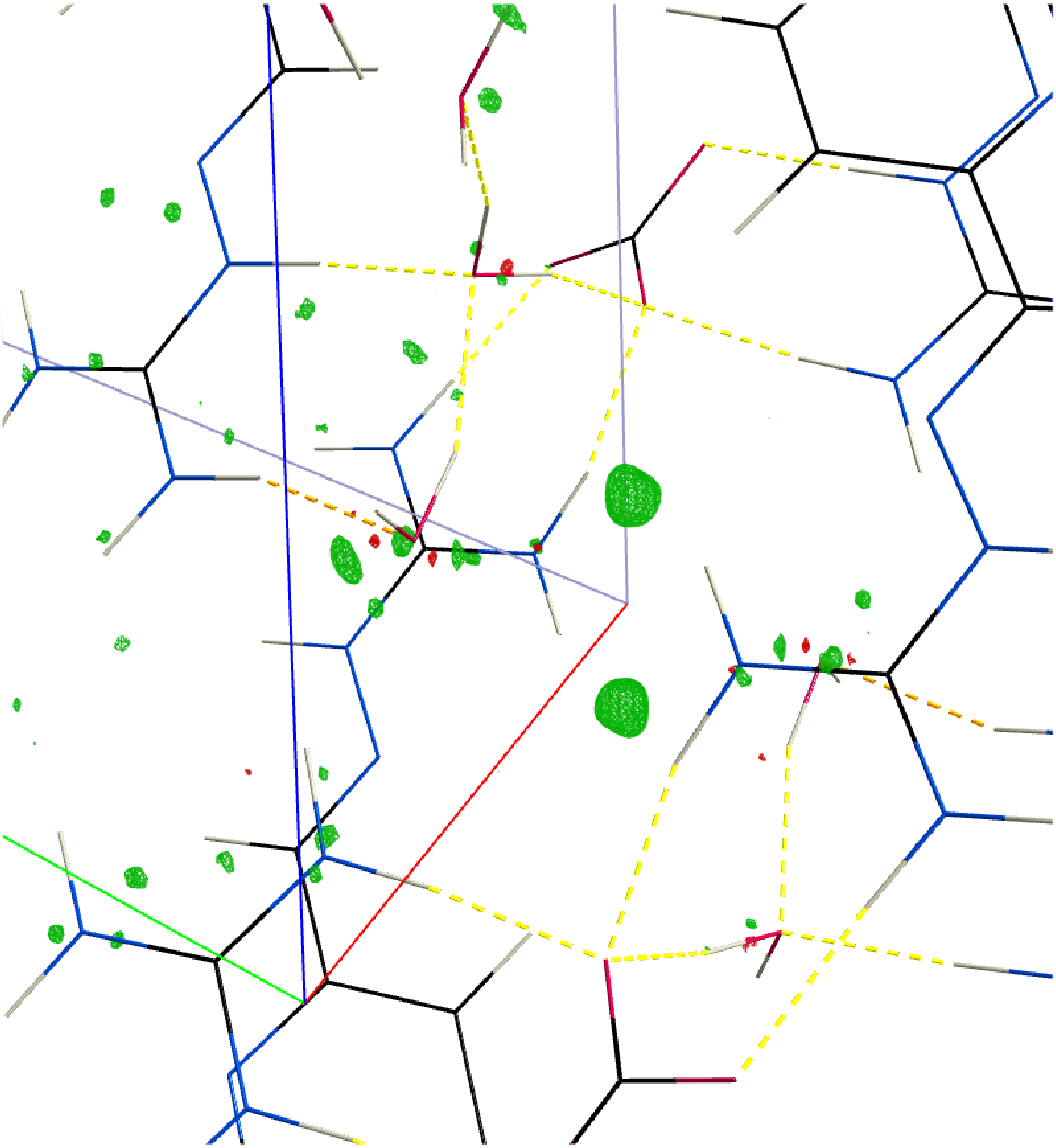

Figure S9. Residual density map from preliminary multipole refinement excluding the partially occupied OH group. The maximum is 0.44 e Å^{-3} while residual density sigma is ~0.04 e Å^{-3} (where sigma is estimated on residual density analysis of the final model).

Maximum coordinates; peak height: 0.9764 -0.0205 0.0792; 0.44 e Å^{-3}

Table S1. Critical point properties of covalent bonds. R_{ij}, d_1 and d_2 (Å); ρ (e Å^{-3}); $\nabla^2\rho$, λ_1, λ_2, λ_3 (e Å^{-5}); g, v, h (au).

Atom 1	Atom 2	R_{ij}	d_1	d_2	ρ	$\nabla^2\rho$	λ_1	λ_2	λ_3	g	v	h	ε	n_{topo}
PyBig														
C1	N1	1.325	0.749	0.576	2.49	-26.40	-21.23	-18.20	13.03	0.3631	-1.0000	-0.6370	0.17	1.26
C1	N2	1.327	0.543	0.785	2.46	-30.35	-22.18	-18.20	10.03	0.3225	-0.9599	-0.6374	0.22	1.16
C1	N3	1.352	0.560	0.792	2.37	-28.05	-20.98	-17.89	10.82	0.3087	-0.9085	-0.5997	0.17	1.10
C2	H2	1.095	0.744	0.350	1.83	-19.78	-18.49	-17.67	16.38	0.1899	-0.5850	-0.3951	0.05	0.89
C2	C3	1.468	0.731	0.737	1.91	-16.05	-14.20	-12.88	11.03	0.2390	-0.6444	-0.4055	0.10	1.08
C2	N4	1.286	0.509	0.778	2.64	-31.84	-23.05	-18.91	10.11	0.3793	-1.0889	-0.7096	0.22	1.34
C3	C4	1.398	0.704	0.694	2.14	-20.07	-16.60	-13.92	10.45	0.2841	-0.7764	-0.4923	0.19	1.26
C3	N5	1.347	0.582	0.766	2.35	-22.86	-19.26	-17.32	13.72	0.3366	-0.9103	-0.5737	0.11	1.17
C4	H4	1.088	0.727	0.361	1.88	-20.42	-18.61	-17.98	16.17	0.1993	-0.6104	-0.4112	0.03	0.93
C4	C5	1.391	0.692	0.699	2.16	-20.02	-16.31	-14.34	10.63	0.2921	-0.7920	-0.4998	0.14	1.31
C5	H5	1.086	0.724	0.363	1.87	-20.25	-18.36	-17.97	16.07	0.1983	-0.6066	-0.4083	0.02	0.93
C5	C6	1.388	0.698	0.690	2.17	-20.32	-16.59	-14.26	10.53	0.2923	-0.7953	-0.5030	0.16	1.30
C6	H6	1.085	0.730	0.355	1.89	-20.85	-18.92	-18.41	16.48	0.2005	-0.6174	-0.4168	0.03	0.92
C6	C7	1.402	0.684	0.718	2.11	-19.48	-16.24	-13.72	10.48	0.2791	-0.7604	-0.4812	0.18	1.26
C7	N5	1.345	0.771	0.574	2.37	-23.75	-19.50	-17.55	13.30	0.3391	-0.9246	-0.5855	0.11	1.18
C7	C8	1.470	0.736	0.734	1.90	-15.87	-14.20	-12.71	11.04	0.2372	-0.6391	-0.4018	0.12	1.08
C8	N6	1.284	0.790	0.494	2.64	-31.87	-22.81	-19.13	10.07	0.3794	-1.0894	-0.7100	0.19	1.34
C8	H8	1.096	0.738	0.358	1.84	-19.56	-18.35	-17.39	16.18	0.1927	-0.5882	-0.3956	0.06	0.91
C9	N7	1.358	0.777	0.581	2.35	-25.50	-20.58	-17.35	12.43	0.3194	-0.9034	-0.5839	0.19	1.12
C9	N8	1.322	0.760	0.563	2.49	-28.67	-22.03	-18.23	11.60	0.3452	-0.9878	-0.6426	0.21	1.21

C9	N9	1.326	0.556	0.770	2.45	-27.40	-21.12	-17.78	11.51	0.3421	-0.9685	-0.6263	0.19	1.21
N1	H12	1.037	0.781	0.256	2.13	-32.37	-29.72	-28.53	25.88	0.1960	-0.7277	-0.5317	0.04	0.69
N1	H11	1.005	0.765	0.240	2.13	-31.95	-30.95	-29.88	28.89	0.2004	-0.7323	-0.5319	0.04	0.64
N2	H22	1.013	0.757	0.257	2.16	-30.16	-29.38	-28.12	27.35	0.2197	-0.7522	-0.5325	0.05	0.73
N2	H21	1.029	0.778	0.251	2.09	-31.28	-29.40	-28.37	26.49	0.1910	-0.7066	-0.5155	0.04	0.66
N3	H3	1.057	0.806	0.252	2.03	-31.38	-28.83	-27.63	25.08	0.1711	-0.6677	-0.4967	0.04	0.63
N3	N4	1.362	0.689	0.673	2.38	-7.05	-20.15	-18.58	31.68	0.4550	-0.9830	-0.5281	0.08	1.38
N6	N7	1.357	0.669	0.688	2.41	-7.88	-20.54	-19.13	31.79	0.4602	-1.0022	-0.5420	0.07	1.37
N7	H7	1.041	0.791	0.250	2.04	-30.49	-29.14	-27.84	26.49	0.1797	-0.6756	-0.4959	0.05	0.62
N8	H82	0.998	0.758	0.240	2.20	-32.86	-31.44	-30.33	28.90	0.2171	-0.7750	-0.5580	0.04	0.69
N8	H81	1.030	0.781	0.249	2.09	-30.96	-29.29	-28.58	26.91	0.1935	-0.7082	-0.5147	0.03	0.66
N9	H92	1.045	0.786	0.259	2.08	-30.10	-28.34	-27.23	25.47	0.1953	-0.7028	-0.5076	0.04	0.69
N9	H91	1.015	0.768	0.247	2.12	-31.53	-30.18	-28.93	27.58	0.2001	-0.7272	-0.5272	0.04	0.66
Carbonate														
C10	O1	1.289	0.475	0.814	2.51	-30.38	-22.95	-20.66	13.23	0.3407	-0.9965	-0.6558	0.11	1.31
C10	O2	1.289	0.476	0.813	2.48	-30.03	-22.64	-20.35	12.97	0.3323	-0.9760	-0.6438	0.11	1.29
C10	O3	1.292	0.491	0.801	2.47	-29.83	-22.36	-19.81	12.34	0.3305	-0.9705	-0.6400	0.13	1.30
Waters														
O4	H42	0.971	0.768	0.203	2.25	-41.79	-38.91	-37.99	35.11	0.1727	-0.7789	-0.6062	0.02	0.55
O4	H41	0.980	0.755	0.225	2.27	-36.72	-36.60	-35.01	34.89	0.2144	-0.8097	-0.5953	0.05	0.59
O5	H52	0.973	0.761	0.212	2.25	-37.03	-36.90	-36.59	36.45	0.2034	-0.7909	-0.5875	0.01	0.53
O5	H51	0.967	0.761	0.205	2.22	-33.49	-36.55	-35.97	39.03	0.2194	-0.7863	-0.5668	0.02	0.45
O6	H62	0.980	0.779	0.201	2.21	-41.44	-38.21	-38.01	34.78	0.1600	-0.7499	-0.5899	0.01	0.53
O6	H61	0.981	0.771	0.211	2.21	-36.63	-36.77	-35.99	36.13	0.1918	-0.7636	-0.5718	0.02	0.51

O7	H72	0.984	0.785	0.199	2.16	-37.75	-37.17	-36.44	35.86	0.1687	-0.7290	-0.5603	0.02	0.47
O7	H71	0.973	0.765	0.208	2.24	-38.56	-37.61	-37.42	36.47	0.1911	-0.7823	-0.5911	0.00	0.52

Table S2. Properties of bond critical points for intermolecular interactions. R_{ij}, d and d_2 (Å); ρ (e Å^{-3}); $\nabla^2\rho$, λ_1, λ_2, λ_3 (e Å^{-5}); g, v, h (au); D_e (kJ/mol).

Atom 1	Atom 2	ORTEP Atom 2	ρ	$\nabla^2\rho$	R_{ij}	d_1	d_2	g	v	h	D_e	λ_1	λ_2	λ_3
O1	H7	46401	0.358	2.09	1.698	1.122	0.577	0.0360	-0.0503	-0.0143	66.0	-2.441	-2.387	6.921
H11	O2	55501	0.354	2.21	1.725	0.596	1.129	0.0364	-0.0499	-0.0135	65.5	-2.312	-2.297	6.817
O3	H91	46401	0.317	2.53	1.725	1.131	0.596	0.0350	-0.0438	-0.0088	57.5	-2.039	-1.960	6.526
O6	H3	65501	0.299	2.47	1.724	1.152	0.572	0.0330	-0.0404	-0.0074	53.1	-2.008	-1.970	6.445
O3	H61	66502	0.296	2.59	1.727	1.134	0.596	0.0336	-0.0403	-0.0067	52.8	-1.875	-1.809	6.275
O2	H81	45401	0.279	2.70	1.751	1.153	0.598	0.0329	-0.0378	-0.0049	49.6	-1.742	-1.713	6.154
O1	H71	56502	0.266	2.17	1.790	1.171	0.619	0.0281	-0.0337	-0.0056	44.2	-1.669	-1.644	5.483
H62	O7	55501	0.258	2.54	1.766	0.594	1.175	0.0300	-0.0337	-0.0036	44.2	-1.657	-1.573	5.774
O2	H41	56502	0.251	2.07	1.798	1.181	0.618	0.0262	-0.0310	-0.0047	40.6	-1.595	-1.579	5.249
O5	H72	65602	0.237	2.56	1.797	1.188	0.611	0.0285	-0.0305	-0.0020	40.0	-1.427	-1.420	5.407
H21	O3	55501	0.244	2.18	1.830	0.635	1.195	0.0264	-0.0302	-0.0038	39.7	-1.489	-1.421	5.089
H42	O6	55501	0.2	2.17	1.868	0.639	1.230	0.0232	-0.0239	-0.0007	31.3	-1.162	-1.149	4.482
O1	H51	56502	0.187	2.29	1.897	1.223	0.676	0.0231	-0.0224	0.0007	29.4	-0.958	-0.943	4.193

H52	N5	55501	0.201	1.61	1.963	0.674	1.290	0.0193	-0.0220	-0.0026	28.8	-1.131	-1.114	3.854
O4	H12	65501	0.154	1.48	2.003	1.291	0.714	0.0155	-0.0156	-0.0002	20.5	-0.814	-0.749	3.037
O7	H82	66602	0.108	1.72	2.115	1.338	0.780	0.0148	-0.0118	0.0030	15.5	-0.481	-0.363	2.561
O4	H92	55401	0.092	1.71	2.129	1.344	0.794	0.0141	-0.0104	0.0037	13.6	-0.405	-0.256	2.369
O7	H6	66602	0.055	0.53	2.561	1.520	1.043	0.0046	-0.0037	0.0009	4.9	-0.191	-0.179	0.897
O5	H5	54501	0.047	0.65	2.647	1.535	1.119	0.0052	-0.0037	0.0015	4.9	-0.141	-0.104	0.893
N1	N3	56502	0.051	0.56	3.256	1.623	1.649	0.0047	-0.0036	0.0011	4.8	-0.122	-0.078	0.763
C3	C9	66602	0.057	0.46	3.280	1.667	1.666	0.0042	-0.0036	0.0006	4.7	-0.097	-0.026	0.586
C7	N6	66602	0.054	0.50	3.267	1.665	1.623	0.0044	-0.0036	0.0008	4.7	-0.071	-0.035	0.604
C3	C7	56602	0.056	0.46	3.295	1.642	1.653	0.0041	-0.0035	0.0006	4.6	-0.075	-0.027	0.562
O4	O8	55601	0.045	0.56	2.131	1.558	0.573	0.0046	-0.0033	0.0013	4.4	-0.257	-0.250	1.072
N3	C9	56602	0.049	0.46	3.398	1.730	1.676	0.0039	-0.0031	0.0008	4.1	-0.050	-0.026	0.534
O5	C5	56602	0.042	0.45	3.216	1.579	1.637	0.0037	-0.0028	0.0010	3.6	-0.084	-0.074	0.612
O7	H4	64501	0.034	0.51	2.807	1.639	1.228	0.0040	-0.0026	0.0014	3.4	-0.074	-0.057	0.643
O6	H4	64501	0.028	0.51	2.807	1.659	1.150	0.0039	-0.0024	0.0015	3.2	-0.073	-0.055	0.641
O1	H8	46401	0.03	0.45	2.796	1.627	1.172	0.0035	-0.0023	0.0012	3.0	-0.088	-0.036	0.577
O4	N2	64501	0.026	0.48	3.366	1.654	1.719	0.0036	-0.0022	0.0014	2.9	-0.076	-0.061	0.615
O8	H22	64501	0.027	0.34	1.738	0.702	1.039	0.0026	-0.0018	0.0009	2.3	-0.130	-0.129	0.596
O4	N2	66502	0.021	0.28	3.550	1.725	1.830	0.0021	-0.0013	0.0008	1.8	-0.047	-0.042	0.366
O6	N8	66602	0.019	0.28	3.535	1.739	1.799	0.0021	-0.0013	0.0008	1.7	-0.046	-0.030	0.359
O7	H8	65602	0.02	0.23	3.138	1.849	1.324	0.0018	-0.0012	0.0006	1.5	-0.053	-0.010	0.297
O1	C5	57502	0.015	0.17	3.867	1.904	1.970	0.0013	-0.0008	0.0005	1.1	-0.012	-0.009	0.187
O4	O8	75502	0.008	0.12	2.876	1.931	0.947	0.0009	-0.0005	0.0004	0.6	-0.023	-0.018	0.159
O1	N2	47502	0.008	0.09	4.214	2.228	2.079	0.0006	-0.0004	0.0003	0.5	-0.010	-0.007	0.104

Table S3-S4. Reflection statistics

Cumulative resolution subsets [s = sin(theta)/lambda , d = 1/(2*s) ; s .le. s_max , d .ge. d_min]

	Nmeasrd	Nunique	<N>	R1	R2	RW	Z	V
d > 10	1	1	1	0	0	0	0	0
d > 8	1	1	1	0	0	0	0	0
d > 6	143	10	14.3	0.0075	0.0098	0.0109	1.092	0.009
d > 4	528	29	18.2	0.0108	0.019	0.0112	1.412	0.014
d > 3.5	834	43	19.4	0.0098	0.0178	0.0105	1.394	0.012
d > 3	1428	70	20.4	0.0191	0.0395	0.0131	1.892	0.024
d > 2.5	2579	121	21.3	0.016	0.0383	0.0123	1.836	0.02
d > 2	4882	226	21.6	0.014	0.0366	0.0121	1.797	0.018
d > 1.5	11964	549	21.8	0.0123	0.035	0.0119	1.66	0.015
d > 1	39636	1836	21.6	0.0136	0.0339	0.0148	1.564	0.017
d > 0.75	84593	4346	19.5	0.0166	0.0337	0.0177	1.391	0.02
d > 0.5	144502	13615	10.6	0.0203	0.0338	0.0197	1.232	0.031
d > 0.4	183602	23751	7.7	0.0226	0.0338	0.0206	1.17	0.039
d > 0.35	189348	25805	7.3	0.0228	0.0338	0.0208	1.165	0.04
d > 0	0	0	0	0	0	0	0	0

Distribution of measured and missing reflections in cumulative fixed resolution subsets [s = sin(theta)/lambda , d = 1/(2*s) ; s .le. s_max , d .ge. d_min]

	n_hkl measured	n_hkl missing	percent completeness
d > 10	1	0	100
d > 8	1	0	100
d > 6	10	0	100
d > 4	29	0	100
d > 3.5	43	0	100
d > 3	70	0	100
d > 2.5	121	0	100
d > 2	226	0	100

d > 1.5	549	0	100
d > 1	1836	0	100
d > 0.75	4346	5	99.9
d > 0.5	13615	1156	92.2
d > 0.4	23751	5356	81.6
d > 0.35	25805	6451	80
d > 0	0	0	0
